# Deletion of a rheumatoid arthritis risk enhancer in mouse disrupts *Bim* expression and immune homeostasis

**DOI:** 10.1016/j.ero.2026.100260

**Published:** 2026-10-17

**Authors:** Stefano Rossi, Kelly S. Hayes, Jake Butler, James Ding, Ellie L. Richards, Antonios Frantzeskos, Gareth J. Howell, Richard K. Grencis, Antony D. Adamson, Stephen Eyre, Gisela Orozco

**Affiliations:** 1 Arthritis UK Centre for Genetics and Genomics, The University of Manchester, Manchester, UK; 2 NIHR Manchester Biomedical Research Centre, Manchester University NHS Foundation Trust, Manchester Academic Health Science Centre, Manchester, UK; 3 Genome Editing Unit, Faculty of Biology, Medicine and Health, The University of Manchester, Manchester, UK; 4 Division of Immunology, Immunity to Infection and Respiratory Medicine, School of Biological Sciences, Faculty of Biology, Medicine and Health, The University of Manchester, Manchester, UK; 5 Lydia Becker Institute of Immunology and Inflammation, University of Manchester, Manchester, UK

## Abstract

**Objectives:**

This study aimed to characterise the *in vivo* function of a murine regulatory region syntenic to the human rheumatoid arthritis (RA)-associated enhancer at the *ACOXL* locus and its role in immunity.

**Methods:**

Using CRISPR-Cas9, we generated the Bim-eΔ mouse model by excising a 15 kb genomic region syntenic to the human RA-associated locus tagged by rs13401811 from C57BL/6J zygotes. *Bim* expression was assessed by quantitative polymerase chain reaction. Thymocytes and splenocytes were profiled by flow cytometry, and whole-spleen RNA-seq was used to define transcriptional changes. Immune responses were tested using a *Trichuris muris* infection model and an antigen-induced arthritis (AIA) protocol.

**Results:**

Bim-eΔ mice show reduced *Bim* expression vs wild-type counterparts. This indicates that *Bim* is a transcriptional target of this region. Enhancer loss was correlated with differences in T cell proportions across age- and sex-stratified cohorts consistent with altered thymic selection. Splenic T and B cells were altered, and the transcriptome skewed toward inflammation. Bim-eΔ and wild-type mice mounted comparable responses to *T. muris*, whereas Bim-eΔ mice developed a severe adverse response during the sensitisation phase of the AIA protocol.

**Conclusions:**

A murine noncoding regulatory region modulates *Bim* expression and immunity *in vivo*. Enhancer deletion altered the splenic transcriptional profile and lymphocyte composition while revealing a context-dependent phenotype upon inflammatory *in vivo* challenge. These findings support a role for RA-associated regulatory architecture in shaping immunity and point to *BIM* regulation as a potential therapeutic lever for restoring immune balance.

**WHAT IS ALREADY KNOWN ON THIS TOPIC**

• The proapoptotic *BCL2L11/BIM* is key in immunity; *Bim* deficiency skews T-cell development and predisposes to autoimmunity in mice. Genome-wide association studies implicate the rheumatoid arthritis (RA)-associated single nucleotide polymorphism rs13401811 in a distal *BCL2L11* enhancer associated with altered enhancer activity and reduced BIM expression in human CD4⁺ T cells.

**WHAT THIS STUDY ADDS**

• CRISPR excision of a murine regulatory region syntenic to the human RA-associated enhancer lowers *Bim* mRNA and is associated with shifts in adaptive immune cell composition across age- and sex-stratified cohorts in Bim-eΔ mouse. Deletion of this regulatory region skews the splenic transcriptome and elicits a marked adverse response during the sensitisation phase of an antigen-induced arthritis model.

**HOW THIS STUDY MIGHT AFFECT RESEARCH, PRACTICE OR POLICY**

• Highlights noncoding regulatory variation as a potential contributor to immune imbalance, emphasising regulatory regions as therapeutic entry points. Supports further investigation of strategies aimed at fine-tuning BIM modulation to regulate immune homeostasis and preserve immune competence.

Alt-text: Unlabelled box dummy alt text

## INTRODUCTION

Genome-wide association studies have linked noncoding variants with risk of autoimmune conditions such as rheumatoid arthritis (RA)—a chronic disorder primarily affecting the joints [1,2]. Many of these genomic variants reside in putative regulatory regions orchestrating cell-type-specific gene expression regarded as enhancers [3]. These elements can physically interact over long distances with target gene promoters and fine-tune their expression [4]. Despite many RA-associated noncoding variants documented [5], the mechanistic insights by which enhancer variation can increase RA risk remain largely unknown. Our group described an RA-associated enhancer located within an intron of the *ACOXL* gene and forming a physical loop with the proapoptotic *BCL2L11/BIM* gene promoter, indicating a direct regulatory mechanism of *BIM* transcription [6,7]. Specifically, this enhancer contains a single nucleotide polymorphism (SNP), rs13401811, whose protective allele is associated with approximately a 40% reduction in chromatin accessibility [6]. As enhancer activity correlates with gene expression levels, reduced activity of the alternative allele implies that carriers may exhibit reduced *BIM* expression. Also, given the role elicited by *BIM* in lymphocyte homeostasis [8], we showed that CRISPR-Cas9 excision of this enhancer in human single-positive (SP) CD4^+^ T cells resulted in a marked decrease in *BIM* expression upon *in vitro* stimulation [6].

Apoptotic molecules, including Bim, preserve immune homeostasis through a regulated elimination of self-reactive cells, a process governed by the interplay of proapoptotic and antiapoptotic proteins, whose dysregulation can promote autoimmune diseases, including RA [[9], [10], [11]]. Ablation of *Bim* expression in mice alters haematopoietic cell frequencies across multiple tissues. Specifically, *Bim*^−/−^ mice display altered T cell development characterised by elevated proportions of thymic SP CD4^+^, CD8^+^, and double-negative (DN; CD4^–^CD8^–^) populations, with a reduced double-positive (DP; CD4^+^CD8^+^) fraction [12]. This phenotype highlights the complex role of BIM in immunity and the importance of understanding how *Bim* expression is transcriptionally regulated to maintain immune cell homeostasis.

Given its role in T-cell biology [12] and previous investigations [6,13], we studied the immunologic impact of the enhancer region regulating *Bim.* We excised the conserved regulatory block syntenic to the human *ACOXL* locus interacting with the *BIM* promoter. This segment is syntenic to the human RA risk locus indexed by rs13396472, rs13401811, and high-linkage disequilibrium (LD) proxy RA variants within this regulatory region [2,6,14]. To assess its function, we generated the Bim-eΔ mouse—a model lacking the murine regulatory region syntenic to the human RA-associated enhancer. Corroborating previous findings [6,13], we report that deletion of the latter region is associated with reduced *Bim* expression, and describe an altered immune cell homeostasis in the thymus and spleen of Bim-eΔ compared with wild-type (WT) animals.

## METHODS

### Ethics statement

Experiments were conducted in accordance with the UK Animal Scientific Procedures Act (1986) under Home Office Project Licence P043A3082 and approved by the Manchester University Animal Welfare and Ethical Board. Animals of both sexes were used throughout the study, as appropriate to the experimental design. The experiments conform to the Animal Research: Reporting of *In Vivo* Experiments (ARRIVE) guidelines.

### Generation of the Bim-eΔ Mouse

The Bim‐eΔ mouse was generated on a C57BL/6J background using CRISPR‐Cas9 gene editing. Single guide RNAs (sgRNAs) targeting flanking sequences of a 15.3 kb murine regulatory region syntenic to the human RA-associated enhancer (2 for the upstream site [g483—ggttacagtccgttaccgcc; g484—tacgttcttcctggcggtaa] and 2 for the downstream site [g324—gtgtgacatgagttgtcact; g325—catgagttgtcactgggatt]) were designed with off‐target activity assessed via the Sanger Institute Genome Editing tool https://wge.stemcell.sanger.ac.uk/ [15], excluding guides with 0 to 2 mismatches and tolerating MM3 guides only if predicted off‐targets were nonexonic. Alt‐R full‐length sgRNAs (IDT) were resuspended in sterile, RNase‐free injection buffer (1 mM Tris‐HCl, pH 7.5; 0.1 mM EDTA). For embryo injection, 1 μg of each sgRNA was complexed with 1 μg of EnGen Cas9 (New England Biolabs) to prepare a final mix of 20 ng/μL each sgRNA and 80 ng/μL Cas9 protein. This mix was microinjected into the pronuclei of fertilised C57BL/6J (Envigo) zygotes following standard protocols. Viable 2‐cell embryos were cultured overnight and transferred into the oviducts of day 0.5 postcoitum pseudopregnant females. Founder mice were identified by genomic DNA extraction from ear clips and polymerase chain reaction (PCR) screening using primers flanking the deletion site (Geno F1: GCTCTGCCACACTTGCTTAG; Geno R2: AACTGCACACGTGAACATGT) which yielded a product only upon successful nonhomologous end joining‐mediated deletion. Deletion-junction amplicons were confirmed by Sanger sequencing, and founder Tg4 was used to establish the colony. The deleted mouse region (chr2:127,756,017-127,771,326; GRCm39) aligns within a conserved syntenic block on human (chr2:110,732,524-111,118,548; hg38) encompassing the selected locus.

### Anatomical assessment and organ measurements

At the experimental endpoint, the Bim‐eΔ and WT mice underwent gross anatomical inspection. Major organs were dissected, rinsed in phosphate-buffered saline (PBS), and weighed using a precision balance. Organ length or size was measured using a sterile ruler.

### Tissue processing

Thymic and splenic tissues were harvested from euthanised mice, mechanically dissociated through 40 μm cell strainers (Corning) to generate single-cell suspensions. Splenic red blood cells were lysed using commercial buffer (Sigma-Aldrich). Cells were maintained, pelleted, and resuspended in complete RPMI-1640 media (cRPMI; 10% fetal bovine serum (FBS), 1% penicillin-streptomycin, 50 μM 2-mercaptoethanol, 1× nonessential amino acids, 25 mM HEPES). Viable cells were determined using 0.4% Trypan Blue (Gibco) exclusion using a haemocytometer or an automated cell counter (Countess II, Thermo Fisher Scientific).

### RNA extraction, quantitative PCR analysis, and library preparation

Total RNA was extracted from thymic homogenates of Bim-eΔ and WT mice using the RNeasy Micro Kit (QIAGEN) as per manufacturer’s protocol. RNA purity and concentration were assessed on a NanoDrop 2000 spectrophotometer (Thermo Scientific). The SuperScript IV VILO Master Mix (Thermo Fisher Scientific) was used to reverse-transcribe a sample-normalised concentration of 35 ng/µL of total RNA to cDNA as per the manufacturer's recommendations. The quantitative PCR (qPCR) experiment was performed on 50 ng of the resulting DNA template using the Luna Universal qPCR Master Mix (New England Biolabs), nuclease-free water, and 0.25 µM forward and reverse target primers (*Bim*, FW: 5’- CCCGGAGATACGGATTGCAC-3’ RV: 5’-GCCTCGCGGTAATCATTTGC-3’; *Hprt*, FW: 5’-TCAGTCAACGGGGGACATAAA-3’ RV: 5’-GGGGCTGTACTGCTTAACCAG-3’). Amplification was performed on a QuantStudio 12K Flex Real-Time PCR System (Applied Biosystems) according to the manufacturer’s protocol. Relative *Bim* expression was calculated using the 2⁻^ΔΔCt^ method [16]. RNA-seq libraries were generated using the Novogene NGS RNA Library Prep Set (PT042) and sequenced on a NovaSeq 6000 (Illumina) using paired-end 150 bp reads, yielding 12 Gb per sample; samples were processed at Novogene.

### RNA-sequencing

The FastQC (v.0.12.1) software was used to check the quality of raw reads, trimmed with Trimmomatic (v.0.39.0) tool for low-quality input [17]. For robustness, reads were pseudoaligned to the *Mus musculus* reference transcriptome (GRCm39) using the Kallisto (v.0.50.1) or Salmon (v.1.10.2) tool [18,19]. Differential expression analysis was performed in R (v.4.3.2) using DESeq2 (v.1.46.0) normalised counts, with significance defined as an adjusted *P* value false discovery rate < 0.05. Significant genes were subsequently ranked by log_2_ fold change [20]. The Ingenuity Pathway Analysis tool (QIAGEN; v.150276282) was used to identify significantly enriched biological pathways using differentially expressed genes as input [21].

### Flow cytometry and reagents

Thymic and splenic cell suspensions were incubated with Zombie UV Fixable Viability Dye (BioLegend) and followed by Fc receptor blocking with anti-CD16/CD32 (clone 93, BioLegend). Next, an equal density of single-cell suspensions (1 × 10^6^ cells/mL) was stained in FACS buffer (PBS supplemented with 2% FBS and 2 mM EDTA) for 30 minutes at 4 °C in the dark and using pretitrated antibody concentrations—as per manufacturers’ recommendations. The following fluorochrome conjugated antibodies were used: anti-CD3 FITC or APC (clone 17A2, Miltenyi Biotec), anti-CD4 Vioblue (clone REA604, Miltenyi Biotec), anti-CD8 BV570 (clone 53-6.7, BioLegend), anti-T cell receptor (TCR)β FITC (clone H57-597, BioLegend), anti-CD19 PE (clone 1D3, BD Biosciences), and anti-CD69 PerCP-Cy5.5 (clone H1.2F3, BioLegend). Apotracker Green (BioLegend) was used to measure the frequency of apoptotic thymocytes and splenocytes—as per manufacturer’s instructions. Samples were acquired on a BD Symphony A5 flow cytometer. Events were recorded using FACSDiva software (BD Biosciences), and data were analysed with FlowJo (BD Biosciences; v.10.10.0).

### Apoptosis assay

Thymocytes and splenocytes from Bim‐eΔ and WT mice were cultured at 1 × 10^6^ cells/mL in cRPMI as previously described but supplemented with 2% FBS and incubated for 18 hours at 37 °C under 5% CO_2_. Apoptosis was measured with the Apotracker Green (BioLegend) reagent and analysed by flow cytometry.

### Trichuris muris (T. muris) infection model and parasite maintenance

WT and Bim-eΔ mice were age- and sex-matched as far as possible (12-16 weeks old; n = 5 per group) and orally gavaged with either a low dose (LoD; 35 eggs in 200 μL dH₂O) or a high dose (HD; 400 eggs in 200 μL dH₂O) of *T. muris* and weighed weekly. All mice were humanely euthanised on day 35 postinfection (p.i.). Stock infections of *T. muris* E isolate (Wellcome Research Laboratories) were maintained in susceptible mouse strains (AKR, SCIDs, or athymic) using standard protocols. Adult worm burdens were manually counted in caeca and proximal colons at day 35 p.i.

### T muris specific antibody analysis

Parasite-specific immunoglobulin (Ig)G1 and IgG2a were measured by capture enzyme-linked immunosorbent assay. Immulon IV plates (Dynatech) were coated with *T. muris* excretory/secretory antigen (5 µg/mL), blocked (3% bovine serum albumin in PBS, 0.05% Tween), and incubated with 8 serial 2-fold dilutions of mouse sera. Parasite-specific antibody was detected using biotinylated rat anti-mouse IgG1 (Serotec Ltd.) and biotinylated rat anti-mouse IgG2a (PharMingen). Plates were read at 405 nm on a VersaMax microplate reader (Molecular devices).

### Histology

Caecal tissue was fixed in 4% paraformaldehyde, paraffin-embedded and sectioned at 5 µm. Sections were mounted on gelatin-coated slides, deparaffinised, rehydrated and stained with periodic acid–Schiff (PAS; Vickers Laboratories Limited) and Mayer’s haematoxylin (Sigma). The slides were dehydrated and mounted with DPX mountant (Raymond A. Lamb Laboratory Supplies). Images were acquired on a 3D-Histech Pannoramic-250 microscope slide-scanner using a 10× objective (Zeiss). Digital images were used to measure goblet cells, crypt length, and muscle wall thickness.

### Antigen-induced arthritis

An antigen-induced arthritis (AIA) protocol was initiated using a methylated bovine serum albumin (methylated bovine serum albumin [mBSA]; Merck Life Science) and complete Freund’s adjuvant (CFA; MD Bioproducts). Anaesthetised mice were sensitised by intradermal mBSA/CFA at the base of the tail at day 1, followed by intraperitoneal administration of pertussis toxin (Merck Life Science), and day 7. Animals progressing to day 21 were challenged intra-articularly with 10 μL mBSA injected into a knee. Clinical signs of arthritis (altered gait, knee swelling) were monitored daily. Mice were humanely culled between days 7 and 31 according to predefined humane endpoints and institutional guidelines.

### Statistical analysis

Statistical analyses were performed using GraphPad Prism (Dotmatics; v.10.5.0). Groups were compared using prespecified pairwise comparisons with unpaired, 2-tailed Student’s or Welch’s *t* test. Age- and sex-stratified cohorts were analysed separately. Flow cytometry and qPCR results are presented as mean ± SD. Each point represents a biological replicate. Significance was set at *P* < .05. For the *T. muris* infection experiments, data are shown as mean ± SEM; worm burden graphs display the mean as a line with individual data points overlaid to illustrate the spread.

## RESULTS

### Development of the Bim-eΔ mouse model

We targeted the human RA locus indexed by rs13396472 containing a single SNP—ie, rs13401811—showing allelic imbalance and acting as a chromatin accessibility quantitative trait locus for the overlapping ATAC-seq peak and *in vitro* evidence [2,6]. These previous human data support rs13401811 as a candidate causal regulatory variant within this RA-associated block. Moreover, the human *ACOXL* locus shows rs13401811 and cognate high-LD proxies within the enhancer region, overlapping T-cell enhancer marks, evolutionary and functionally conserved in mouse; this region is more extensive in our Bim-eΔ allele than in the E*^BAB(Bub1-Acoxl-Bim)^* model [13] described by Hojo et al [13] (Supplementary Fig S1). To test the *in vivo* consequence of perturbing this RA-related regulatory element, we deleted an intronic enhancer block syntenic to the human regulatory region containing rs13401811. Therefore, we designed 2 sgRNAs flanking the block to mediate the excision (Fig 1A). Sanger sequencing of the amplicons confirmed the enhancer deletion in each founder, consistent with nonhomologous end joining–mediated repair following Cas9-induced double-strand breaks (Fig 1B). To genotype founders, we used PCR with primers RG12 Geno F1 and Geno R2, which amplify a product only when the CRISPR-mediated deletion brings their binding sites, ordinarily >15 kb apart in WT animals, into proximity. PCR screening identified 3 mice with expected amplicons and a colony was established using Pup #Tg4 (Fig 1C). qPCR on thymocytes from Bim-eΔ mice showed a marked reduction of *Bim* transcripts relative to WT controls—supporting *Bim* as a transcriptional target of the deleted regulatory region (Fig 1D).

**Figure 1:**
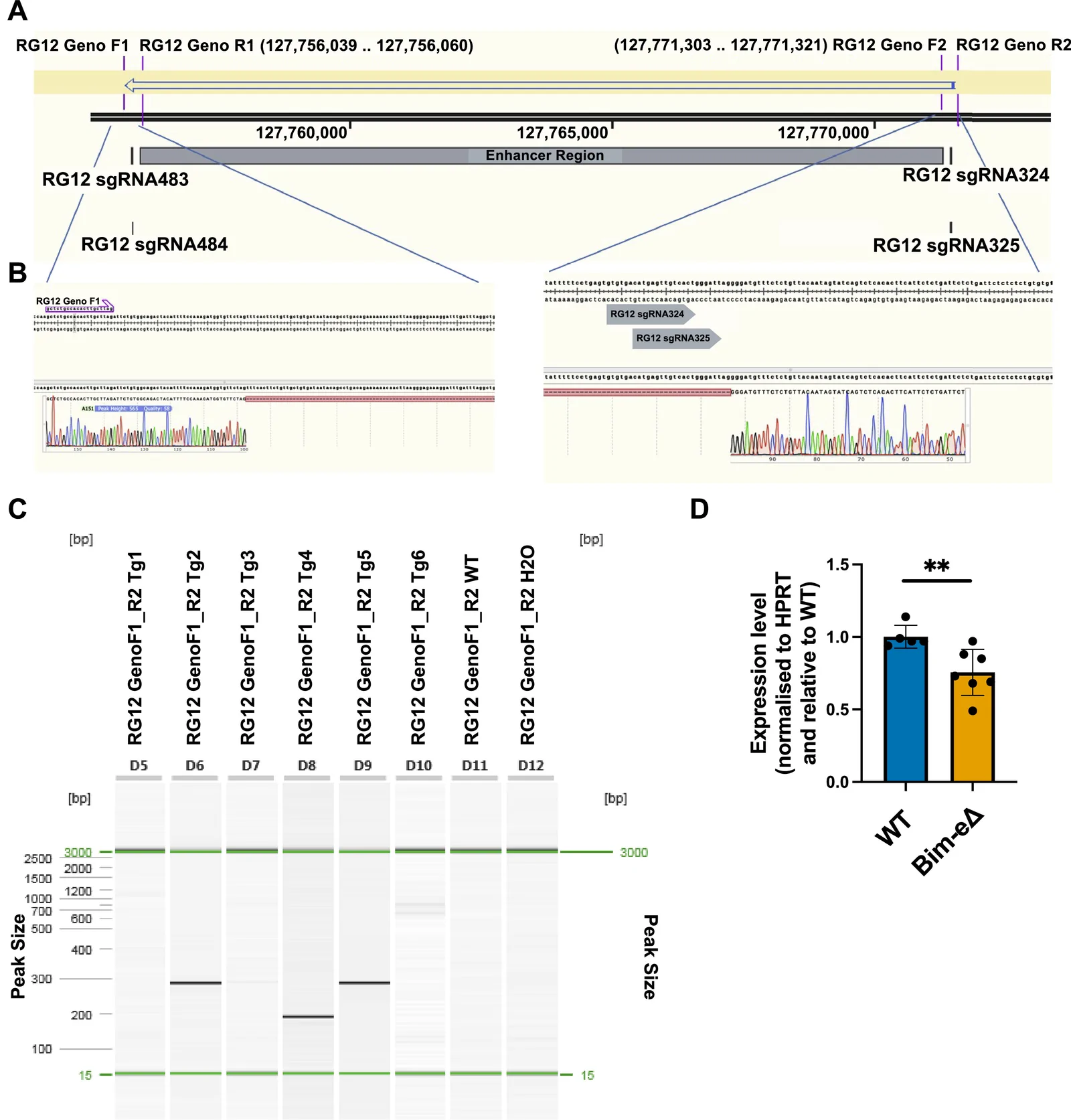
CRISPR-Cas9 mediated deletion of the *Bim* enhancer in C57BL/6J mice, generating the Bim-eΔ mouse model. (A) Schematic indicating the primer binding sites flanking the targeted deletion region generated via nonhomologous end joining, yielding a PCR product. (B) Sanger sequencing traces of the amplicon(s) confirming the precise deletion event. (C) PCR analysis of genomic DNA from founder mice using Geno F1 and Geno R2 primers, demonstrating successful deletion in selected founders. (D) Bar graph indicating qPCR analysis of *Bim* in thymocytes from WT and Bim-eΔ mice where expression level is normalised to *HPRT*. Horizontal lines show the mean ± SD. Each symbol denotes an independent biological replicate. **P < .01 (unpaired 2-tailed Welch’s *t* test; N = 5-7). PCR, polymerase chain reaction; qPCR, quantitative polymerase chain reaction; WT, wild-type.

### The Bim-eΔ mouse model shows thymic T cell alterations across age- and sex-stratified cohorts

To explore the impact of enhancer deletion on thymic T cell development in our murine model, we compared thymic CD4 and CD8 cell proportions in young (7-12 weeks) and middle‐aged (7-12 months) Bim‐eΔ mice using sex‐matched cohorts. In females and males, Bim‐eΔ mice showed a significantly increased proportion of CD4⁺ and CD8⁺ T cells (Fig 2A-C,F-H) with a significant reduction in the DP fraction in young and middle‐aged females, with a less pronounced decrease in middle‐aged males (Fig 2A,D,F,I). Also, the proportion of DN T cells was altered only in young male mice (Fig 2A,E,F,J). These findings indicate that deletion of the enhancer regulating *Bim* expression is associated with altered T cell development, with differences most pronounced in young mice and attenuated in older cohorts.

**Figure 2:**
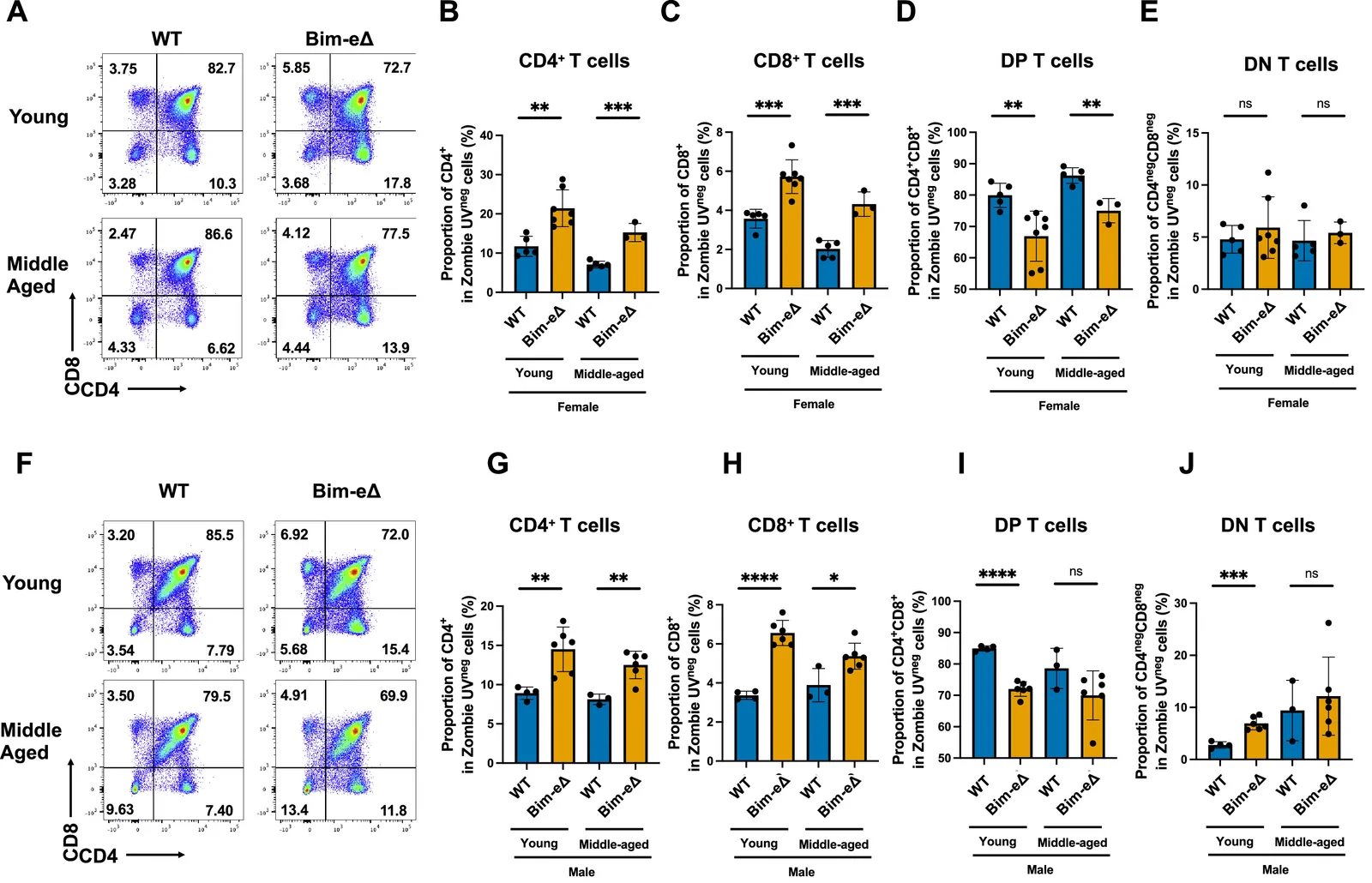
Bim‐eΔ mice show thymic T-cell alterations across age- and sex-stratified cohorts. (A,F) Representative flow plots of thymic CD4 vs CD8 in young (7-12 weeks) and middle‐aged (7-12 months) WT and Bim‐eΔ female (A) and male (F) mice. (B-E), (GJ) Bar graphs showing the proportions of CD4⁺ (B,G), CD8⁺ (C,H), DP (D,I), and DN (E,J) T cells in young and middle‐aged animals. Horizontal lines represent the mean ± SD; each symbol denotes an independent replicate. n.s., not significant (*P* ≥ .05); *P < .05, **P < .01, ***P < .001, ****P < .0001 (unpaired 2-tailed Student’s *t* test; N = 3-7). DN, double negative; DP, double positive; WT, wild type.

### The Bim-eΔ enhancer loss is associated with altered thymic selection, T-cell homeostasis, and apoptosis

To corroborate the hypothesis that thymic development is altered following enhancer deletion, we measured the proportion of thymocytes expressing TCRβ—indicative of cells that have successfully undergone β selection [22]—and cells coexpressing TCRβ and CD69—regarded as postselection thymocytes [23], in young and middle-aged, sex-matched mice. Across the age- and sex-stratified cohorts, Bim‐eΔ mice show an increased proportion of TCRβ^+^ cells and postselection thymocytes accumulating in the thymus (Fig 3A-F). Therefore, we assessed TCRβ and CD69 expression across thymocyte subsets. DP T cells showed a significant increase in postselection thymocytes in Bim‐eΔ (Fig 3G-I). DN, CD4^+^, and CD8^+^ T-cell populations displayed only modest, lineage-specific shifts, and TCRβ^+^ DN T-cell subset was largely unaffected (Supplementary Fig S2). These data suggest that deletion of the *Bim*-associated regulatory region is associated with altered thymic selection and an accumulation of postselection thymocytes [13]. Because BIM controls apoptosis, we next assessed the proportion of apoptotic cells using the Apotracker Green marker and determined which cellular subset contributed most prominently to the apoptotic signal. Our data indicate that the DP T-cell compartment is the primary source of the altered apoptotic profile (Fig 3J,K; Supplementary Fig S3). Collectively, these findings show that the altered proportion of postselection thymocytes within the DP population occurs alongside an altered apoptotic profile upon deletion of the *Bim*-associated regulatory region. Overall, these results indicate that deletion of the *Bim* enhancer is correlated with reduced apoptosis within the DP subset, an accumulation of TCRβ^high^CD69^high^ postselection cells, and a shift in the thymic compartment toward CD4⁺ and CD8⁺ T cells (Figs 1-3).

**Figure 3:**
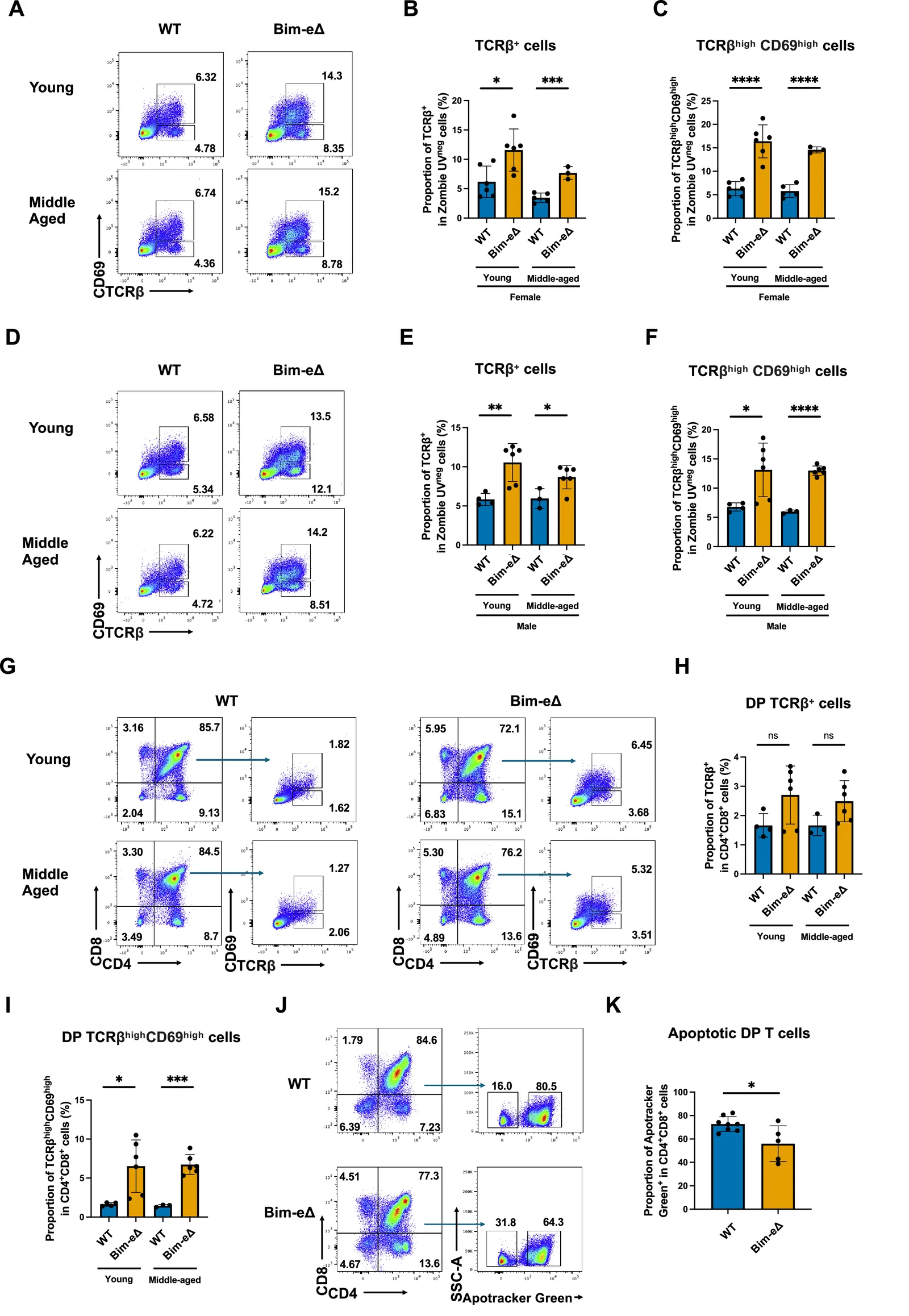
Bim‐eΔ mice show altered thymic selection-associated phenotypes and apoptosis. (A,D) Representative flow plots of thymic TCRβ vs CD69 expression in young (7-12 weeks) or middle-aged (7-12 months) female (A) and male (D) WT and Bim‐eΔ mice. (B,E) Bar graphs illustrating the total percentage of TCRβ^+^ thymocytes in female (B) and male (E) mice (N = 3-6). (C,F) Bar charts depicting the proportion of TCRβ^high^CD69^high^ thymocytes in female (C) and male (F) animals. (G) Representative flow plots of TCRβ vs CD69 expression within the DP compartment in WT and Bim‐eΔ mice, in a sex-matched experiment. (H,I) Bar graphs quantifying the proportion of TCRβ^+^ (H) TCRβ^high^CD69^high^ (I) cells in the thymus across young and middle‐aged cohorts, within the DP T-cell compartment (N = 3-6). (J) Representative figure showing a flow cytometry analysis indicating Apotracker Green^+^ cells within DP T-cell subset, in a sex-matched experiment. (K) Bar graph showing distribution of Apotracker Green^+^ cells within the DP T-cell compartment, denoting apoptotic cells DP thymocytes (N = 5-8). Horizontal lines indicate the mean ± SD; each symbol represents an independent replicate. n.s., not significant (*P* ≥ .05); *P < .05, **P < .01, ***P < .001, ****P < .0001 (unpaired 2‐tailed Student’s *t* test). DP, double positive; TCR, T-cell receptor; WT, wild type.

### The Bim‐eΔ mouse model shows an altered splenic immune composition across age- and sex-stratified cohorts

To investigate the effects of enhancer deletion in peripheral immunity, we analysed splenic cell populations. In contrast to the E*^BAB^* model [13], young Bim‐eΔ mice showed an altered immune profile (Fig 4). Specifically, the frequencies of CD3^–^CD19^–^ (non‐T/B cells) and CD19^+^ splenocytes (B cells) were reduced in young Bim‐eΔ mice relative to controls, whereas no differences were observed in middle‐aged mice (Fig 4A-C). This attenuated phenotype in older Bim‐eΔ animals may reflect age‐related physiological changes [24] or the less pronounced impact of this enhancer at the periphery. Importantly, Bim‐eΔ mice show an increased fraction of T cells compared with WT animals (Fig 4A,D). Within the T-cell compartment, DN and CD4⁺ T cells significantly decrease in Bim‐eΔ mice compared with control animals in both female and male cohorts, with middle‐aged males showing no change in CD4⁺ frequencies (Fig 4E-G,I-K). In both females and males, we measured a higher frequency of CD8⁺ T cells in young Bim‐eΔ mice (Fig 4E,H,I,L). Although females maintain this increase in the middle-aged cohort, CD8⁺ cell frequencies remain unchanged in middle‐aged males (Fig 4I-L). These results indicate that enhancer deletion correlates with an altered splenocyte distribution across the age- and sex-stratified cohorts, implying an alteration of baseline immune homeostasis.

**Figure 4:**
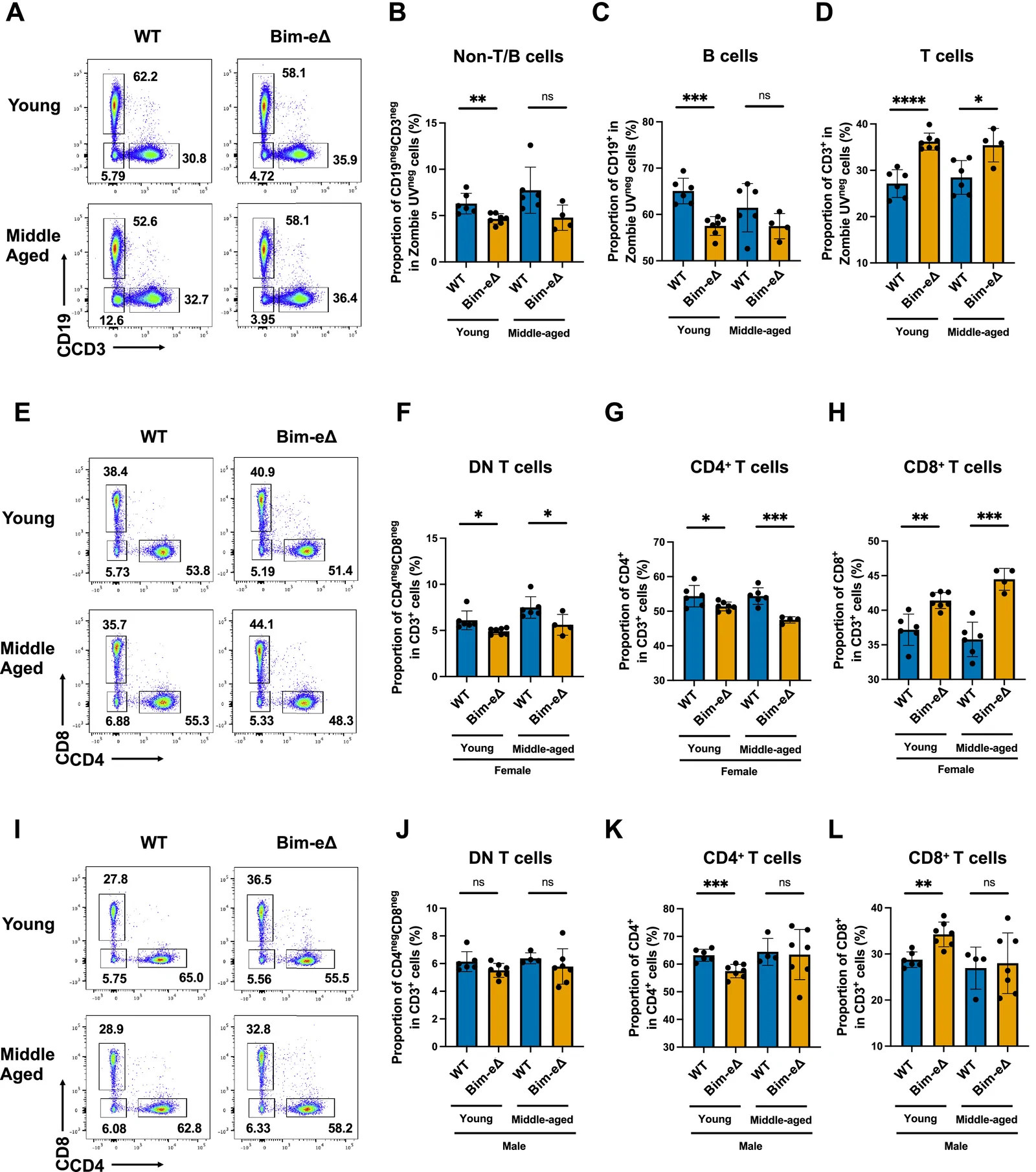
Baseline splenic immune homeostasis is altered in Bim‐eΔ mice. **(**A) Representative plot of CD3- vs CD19-stained splenocytes in young (7-12 weeks) or middle-aged (7-12 months) WT and Bim‐eΔ mice, in sex-matched experiments. (B-D) Bar graphs showing the proportions of non-T/B (B), B (C), T (D) cells in young (7-12 weeks) and middle‐aged (7-12 months) Bim‐eΔ animals. (E,I) Representative flow cytometry plot figures identifying, within the CD3^+^ subset, proportion of DN (F,J), CD4^+^ (G,K), CD8^+^ T (H,L) cells expression in young and middle-aged WT and Bim‐eΔ sex-matched animals (E-H, female; I-L, male). Horizontal lines represent the mean ± SD; each symbol denotes an independent replicate. n.s., not significant (*P* ≥ .05); *P < .05, **P < .01, ***P < .001 (unpaired 2-tailed Student’s *t* test; N = 4-7). DN, double negative; WT, wild type.

### Bim‐eΔ mice show an altered frequency of splenic TCRβ^+^ cells

To determine if deletion of the *Bim*-associated regulatory region correlated with an altered splenic T-cell phenotype, we measured splenic TCRβ^high^CD69^high^ and TCRβ^+^ cell fractions in male and female Bim‐eΔ mice at different ages. In both female and male young cohorts, mice exhibit a significant increase in the TCRβ^+^ population, with no differences observed in the TCRβ^high^CD69^high^ subset (Fig 5A-F). To further elucidate the T-cell subpopulation contributing most to this increase, we assessed TCRβ expression across individual T-cell subsets. Our results indicate that only DN T cells showed an increase in TCRβ in young and middle‐aged Bim‐eΔ mice compared with WT controls (Fig 5G-H, Supplementary Fig S4). These results suggest that *Bim*-associated regulatory region deletion is associated with an increased proportion of TCRβ^+^ cells, predominantly driven by a higher percentage of the DN T-cell compartment. This and previous results indicate that, within the DN T-cell population, there is an enrichment for TCRβ-expressing cells.

**Figure 5:**
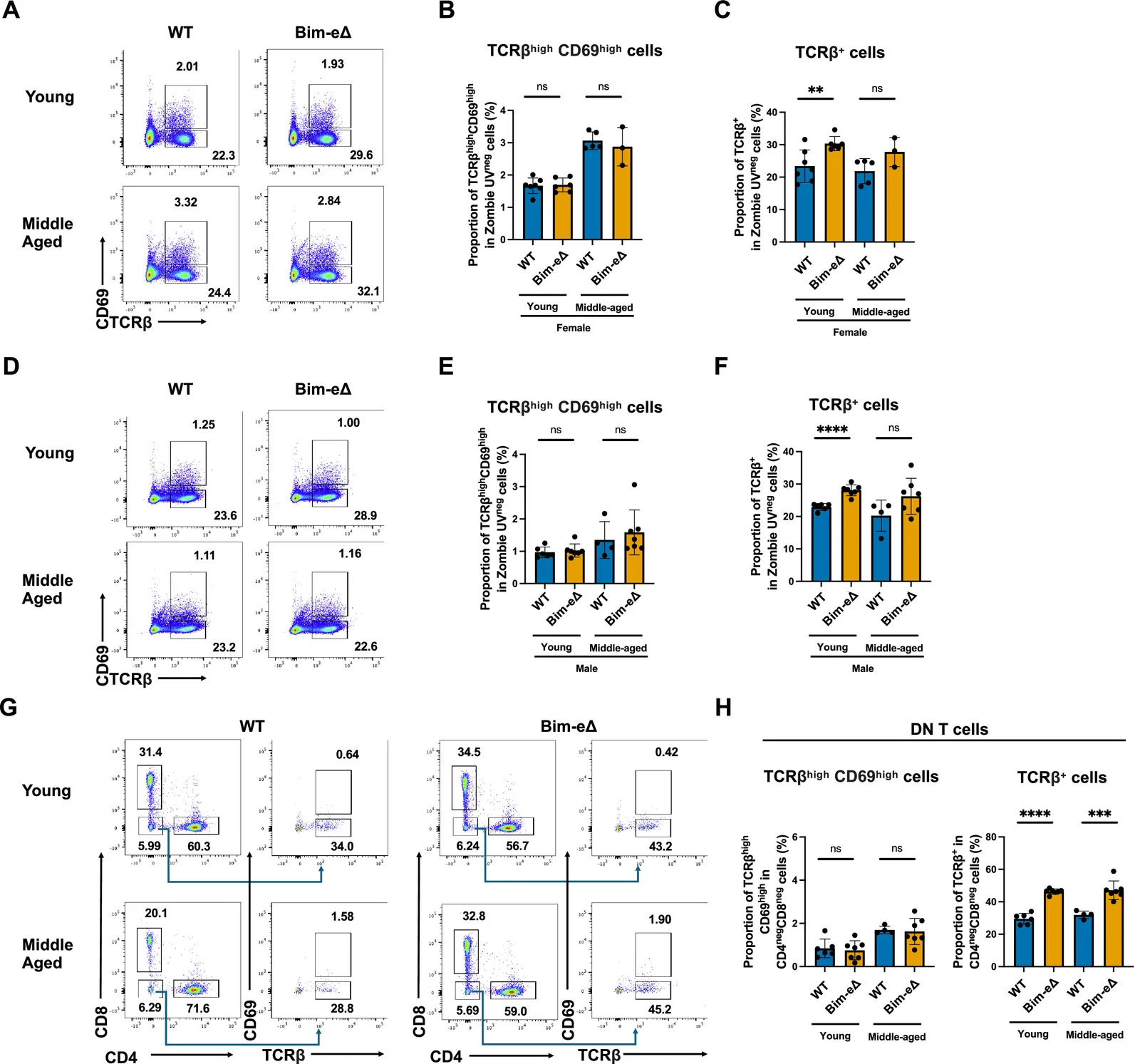
Deletion of the *Bim*-associated regulatory region alters the frequency of splenic TCRβ^+^ cells in Bim-eΔ mice. A. Representative flow cytometry analysis of TCRβ and CD69 expression on splenocytes from young and middle‐aged WT and Bim‐eΔ female (A-C) and male (D-F) mice. (B, E) Bar graphs showing the proportions of TCRβ^high^CD69^high^ cells. (N = 3-7). (C, F) Bar plots representing the overall percentage of TCRβ⁺ splenocytes. (G) Flow cytometry analysis illustrating the gating strategy used to identify DN T cells. H. Bar graph of the proportion of TCRβ⁺ cells within the DN T-cell subset in WT and Bim‐eΔ mice, stratified by age. Horizontal lines represent the mean ± SD; each symbol denotes an independent replicate. n.s., not significant (*P* ≥ .05); *P < .05, **P < .01, ***P < .001, ****P < .001 (unpaired 2‐tailed Student’s *t* test; N = 4-7). DN, double negative; TCR, T-cell receptor; WT, wild type.

### Bim‐eΔ mice show an altered splenic transcriptional profile compared with WT counterparts

We performed transcriptomic analysis of spleen to identify key pathways and target genes affected by enhancer deletion. The principal component analysis plot revealed 2 distinct clusters corresponding to WT and Bim‐eΔ mice (Fig 6A). Differential expression analysis identified 588 genes, including upregulation of *Versican* [25], *Ccl2* [26], *Clec5a* [27], *Nlrp12* [28], and *Chi3l1* [29], which play crucial roles in immune homeostasis and downregulation of *CD209c* [30], *C4a* [31], and *Gpr21* [32] (Fig 6B, Supplementary Fig S5). Pathway analysis corroborated this finding, showing enrichment of pathways associated with inflammation, immune responses, and apoptosis (Fig 6C). Collectively, these splenic transcriptional changes in Bim‐eΔ relative to controls reflect a general disruption of immune homeostasis at baseline.

**Figure 6:**
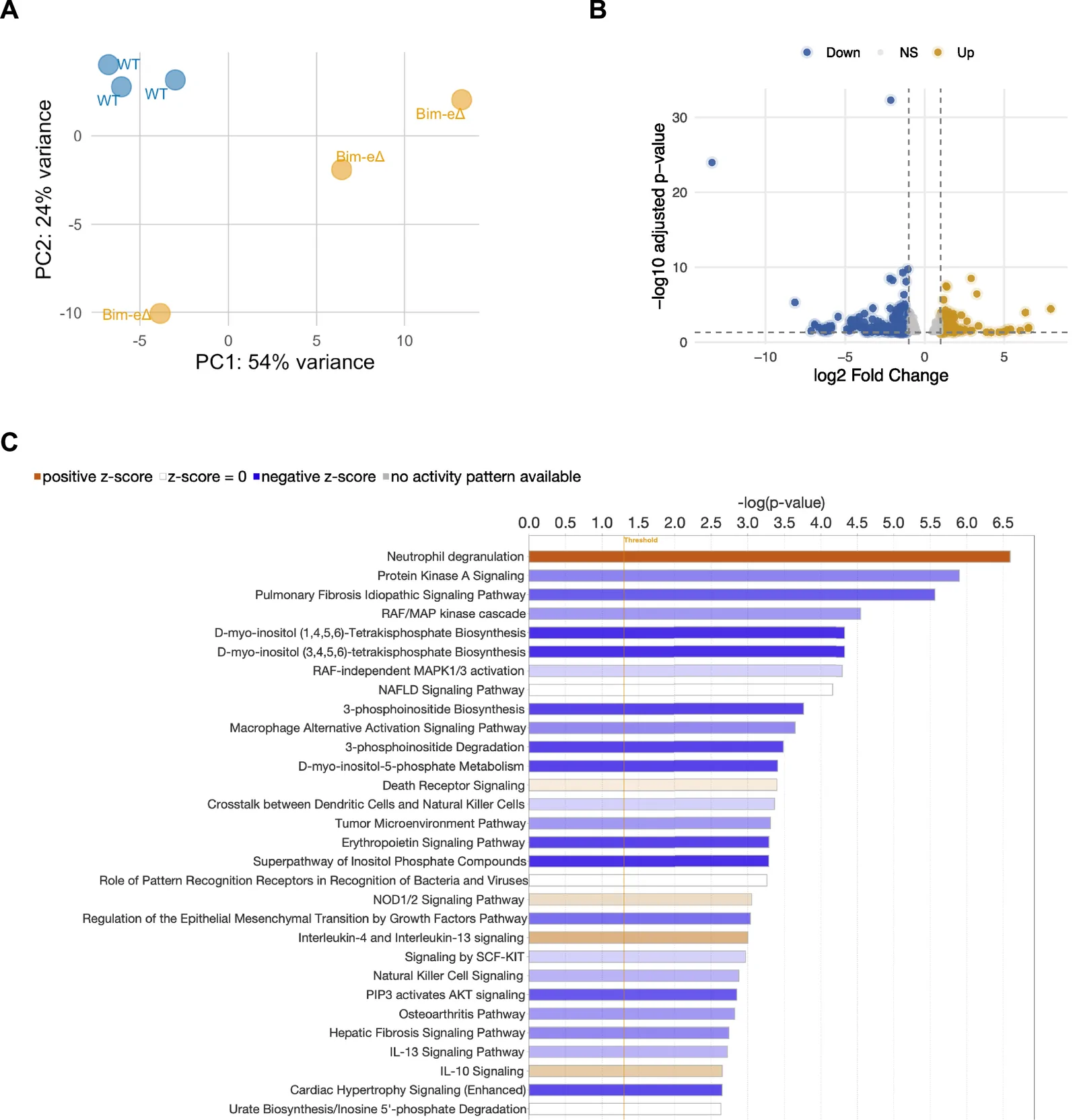
Upon deletion of the *Bim*-associated regulatory region, transcriptomic analysis reveals baseline splenic immune perturbations. (A) PCA plot displays clustering of Bim‐eΔ (in yellow) and control mice (in blue) samples where each point represents a biological replicate, positioned according to PC1 and PC2, accounting for the highest source of experimental variance. (B) Volcano plot showing differentially expressed genes between Bim‐eΔ and WT mice (padj < 0.05). The 240 genes upregulated in the Bim‐eΔ relative to WT condition (LFC > 0) are shown in yellow, and the 348 downregulated genes (log fold change < 0) are shown in blue. (C) Ingenuity pathway analysis (IPA) results show the top 30 most significantly changed pathways in the dataset, with an IPA-predicted activation (orange), inhibition (blue), or no predicted activity (white), and statistical significance expressed as –log_10_(*P* value) (N = 3). IPA, ingenuity pathway analysis; PCA, principal component analysis; TCR, T-cell receptor; WT, wild type.

### Bim‐eΔ mice mount normal immune responses to T. muris and show a marked adverse response during AIA sensitisation

We next asked whether baseline immunological alterations in Bim-eΔ mice translated into measurable effects *in vivo*. Therefore, Bim‐eΔ mice and WT controls received LoD or HD *T. muris*, a helminth infection model to induce chronic (Th1-driven) or acute infection (Th2-driven), respectively [33]. Body weight and worm burdens did not differ between genotypes in either condition (Fig 7A,B), indicating preserved control of parasite load. We measured parasite‐specific IgG1 and IgG2a—proxies for type 2 (IL-4-driven) and type 1 (interferon-γ-driven) responses, respectively. Although IgG1 responses were similar in WT HD and LoD mice (Fig 7C), IgG2a responses were higher in LoD mice than HD mice (Fig 7D). No differences between genotypes were observed, indicating intact humoral immunity in our model. Caecal crypt length, muscle wall thickness, and goblet cell number did not change across groups (Fig 7E-H), consistent with the absence of baseline clinical differences (Supplementary Fig S6). Thus, the *Bim*-associated regulatory region did not alter caecal histopathology in this infection model. We next assessed whether the altered baseline immune homeostasis observed could be further revealed upon an inflammatory *in vivo* challenge. Therefore, WT and Bim-eΔ mice were subjected to the AIA protocol. Preliminary data indicate that, in contrast with WT animals that completed the full immunisation and intra-articular challenge regimen, Bim-eΔ mice developed a marked adverse response during sensitisation, including marked skin changes with patchy alopecia at the injection site, indicative of local inflammation, and reached humane endpoints by days 7 to 8 (n = 3; Supplementary Fig S7). Therefore, Bim-eΔ mice did not progress to the intra-articular challenge with mBSA, precluding assessment of arthritis severity and indicating altered response to the inflammatory sensitisation regimen.

**Figure 7:**
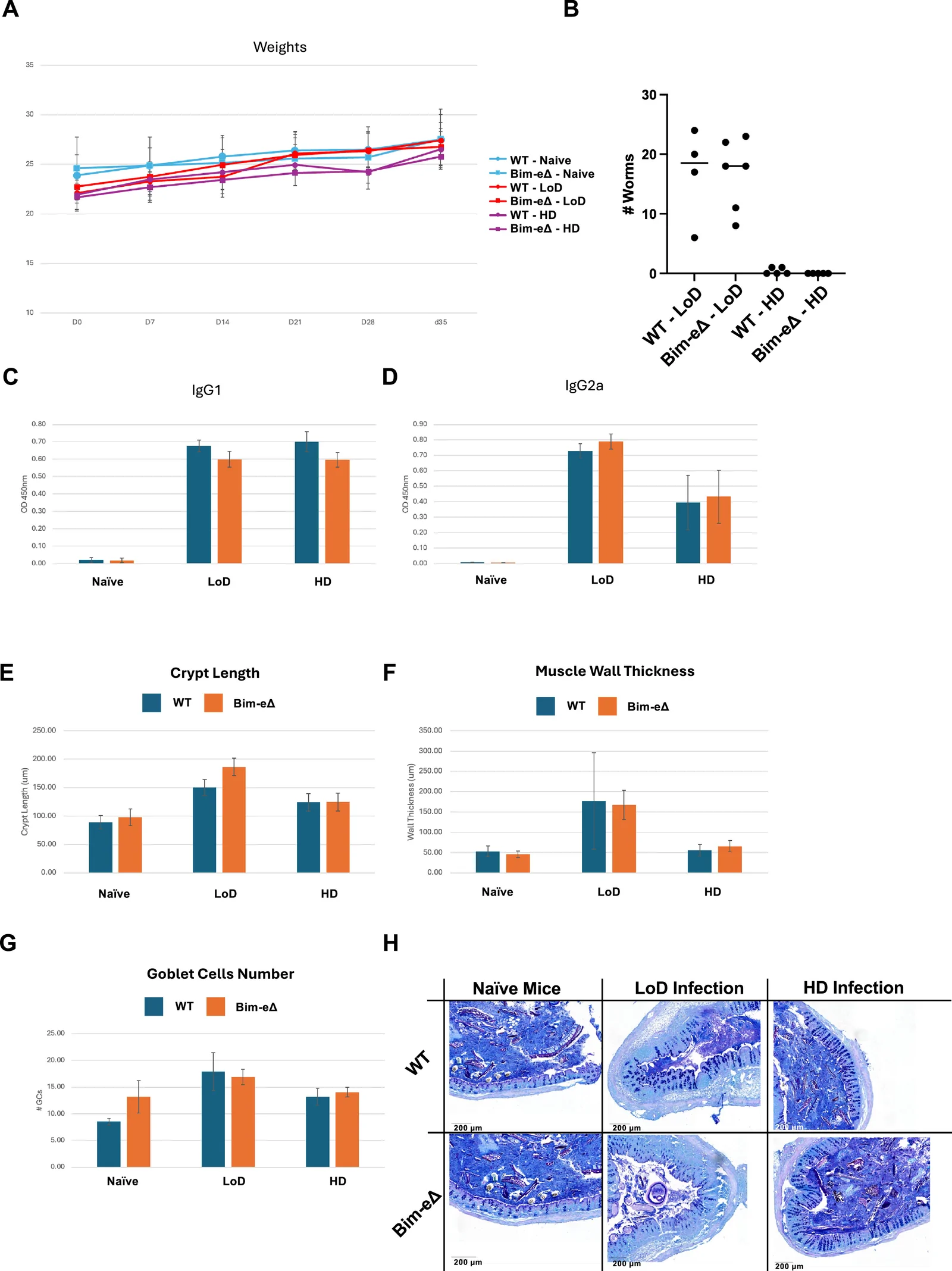
*Bim* enhancer deletion does not affect parasitic immunity or caecal histopathology in *T. muris* infection. (A) Line graph depicting weight change (grams) over days postinfection for HD, LoD, and control treatments in Bim‐eΔ and WT mice. (B) Dot plot representing intestinal worm counts following HD and LoD infections in both genotypes. (C) Bar plot of parasite-specific IgG1 titres measured in mice subjected to HD, LoD, or control infection. (D) Bar plot of parasite-specific IgG2a titres measured in mice under HD, LoD, or control conditions. (E-G) Bar plots showing 3 parallel histological measurements from PAS-stained caecal tissue: crypt length (E), muscle wall thickness (F), and goblet cell number (G). (H) Representative histological section of caecal tissue PAS staining illustrating tissue modifications in Bim‐eΔ and WT mice under negative control, LoD, and HD infection conditions (N = 5). Data are presented as mean ± SEM. In the worm burden graph, the line indicates the mean and individual data points are shown to illustrate the spread. HD, high dose; LoD, low dose; PAS, periodic acid–Schiff; WT, wild type.

## DISCUSSION

Alterations in BIM-mediated apoptosis are linked to autoimmune diseases, including RA [10], type 1 diabetes [34] and systemic lupus erythematosus [35]. This study shows differences across age- and sex-stratified cohorts upon excision of a *Bim*-associated regulatory region using CRISPR-Cas9. It builds on previous findings from our group [6] where an enhancer containing RA-associated SNPs, located in the intronic region of a gene (*ACOXL*), modulates the expression of a distant genetic unit. This underscores the importance of noncoding genomic regions in modulating immunity, providing novel insights into how enhancer deletion can regulate immunity across different age- and sex-stratified cohorts. It is widely documented that sex hormones modulate genomic enhancer activity in immune cells, influencing gene expression [36]. Moreover, evidence highlights age-related alterations in immunity—characterised by an enhanced innate immune response (inflammaging) associated with physiological thymic involution [37]. A limitation of this study is that the observed reduction in *Bim* mRNA was not validated at the protein level.

Importantly, *Bim*^−/−^ mice show exacerbated inflammation in a serum transfer arthritis model relative to controls and reduced caspase-3 activation [10]. Also, impaired apoptosis was correlated with exacerbated inflammation in a rat AIA model [38]. Our preliminary attempts to induce arthritis via the AIA protocol in Bim‐eΔ were discontinued because severe skin reactions within the first week necessitated humane euthanasia before intra-articular injection, preventing assessment of arthritis severity. This result is consistent with altered response during the sensitisation phase of the procedure. Similar alterations in inflammatory resolution following *Bim* ablation have been described in preclinical models. Wang et al*.* [39] documented delayed resolution of inflammation and increased scarring phenotype in a laser-induced choroidal neovascularisation model.

DP thymocytes are the primary subset exhibiting altered apoptosis, which may account for the increased frequency of SP cells observed in Bim-eΔ. Under physiological conditions, DP cells receiving appropriate TCR signals upregulate CD69, progress through positive selection and differentiate into SP cells, whereas cells failing selection or receiving negatively selecting signals are eliminated. In Bim-eΔ, the altered apoptotic profile together with a larger proportion of TCRβ^high^CD69^high^ DP cells is consistent with perturbed thymic selection. Future studies will be required to determine whether high-affinity or autoreactive clones are retained. The increased thymic TCRβ^+^ cell compartment supports an altered selection process during T-cell development, potentially resulting in an aberrant distribution of TCRβ^+^ thymocytes in Bim-eΔ animals.

At the periphery, inflammatory markers transcriptionally altered reflected broader changes in the splenic microenvironment. Relative to controls, Bim-eΔ mice show an increased proportion of TCRβ^+^ cells within the DN T-cell compartment. Elevated frequencies of TCRβ⁺ DN T cells have been linked to various autoimmune and inflammatory disorders [40]. Importantly, in autoimmune lymphoproliferative syndrome, an increase in TCRβ⁺ DN T cells is associated with defective Fas-mediated apoptosis [41], suggesting that the *Bim*-associated regulatory region may similarly influence DN T-cell homeostasis.

Overall, deletion of the syntenic regulatory block corresponding to the human RA-associated region leads to a measurable reshaping of immune homeostasis *in vivo*. These findings support a role for regulatory variation at this locus in orchestrating immune equilibrium and establish the Bim-eΔ mouse as a useful model to dissect how noncoding risk architecture at the *ACOXL-BIM* locus may influence tolerance-associated processes and inflammatory potential relevant to RA.

## Competing interests

The authors declare they have no competing interests.

## Appendix Supplementary materials

Image, application 1

## Footnotes

Supplementary material associated with this article can be found in the online version at doi:10.1016/j.ero.2026.100260.
