# Supplementary material for: Deletion of a rheumatoid arthritis risk enhancer in mouse disrupts Bim expression and immune homeostasis
Source: EULAR Rheumatol Open. 2026 Oct 17;2(4):100260. doi: 10.1016/j.ero.2026.100260 (PMC13696349; doi:10.1016/j.ero.2026.100260)

Supplementary Figure 1

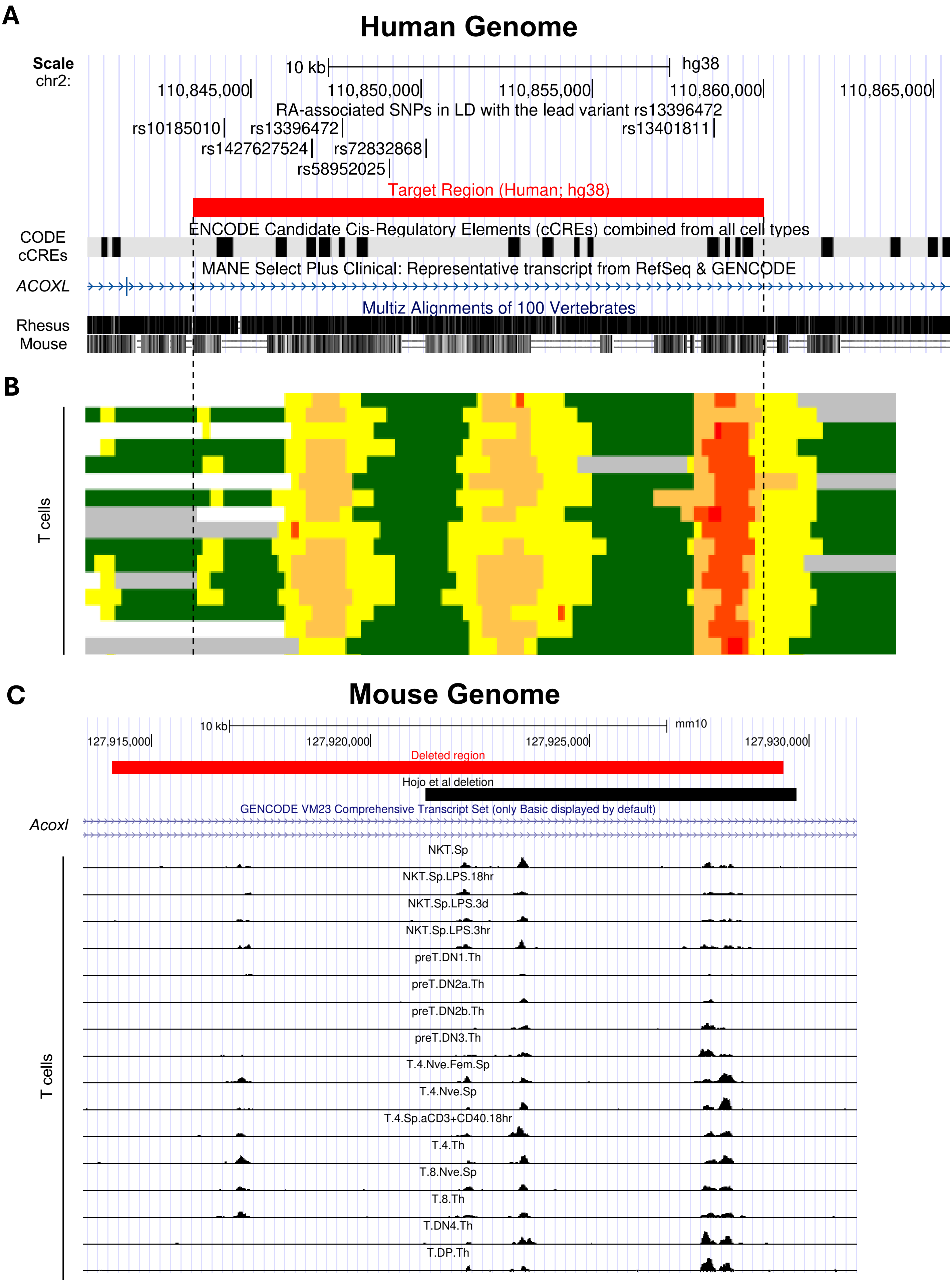

Supplementary Figure 2

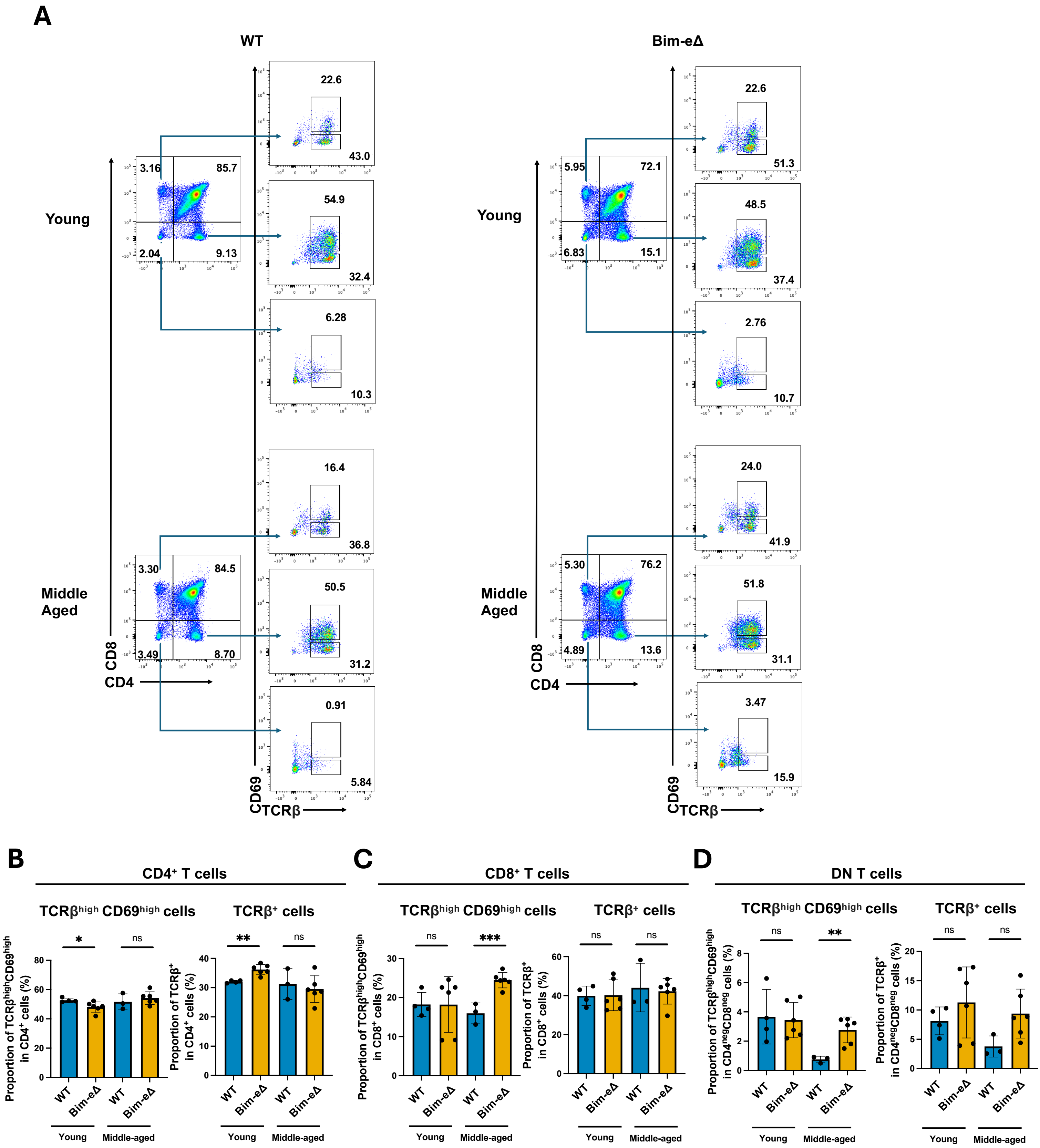

Supplementary Figure 3

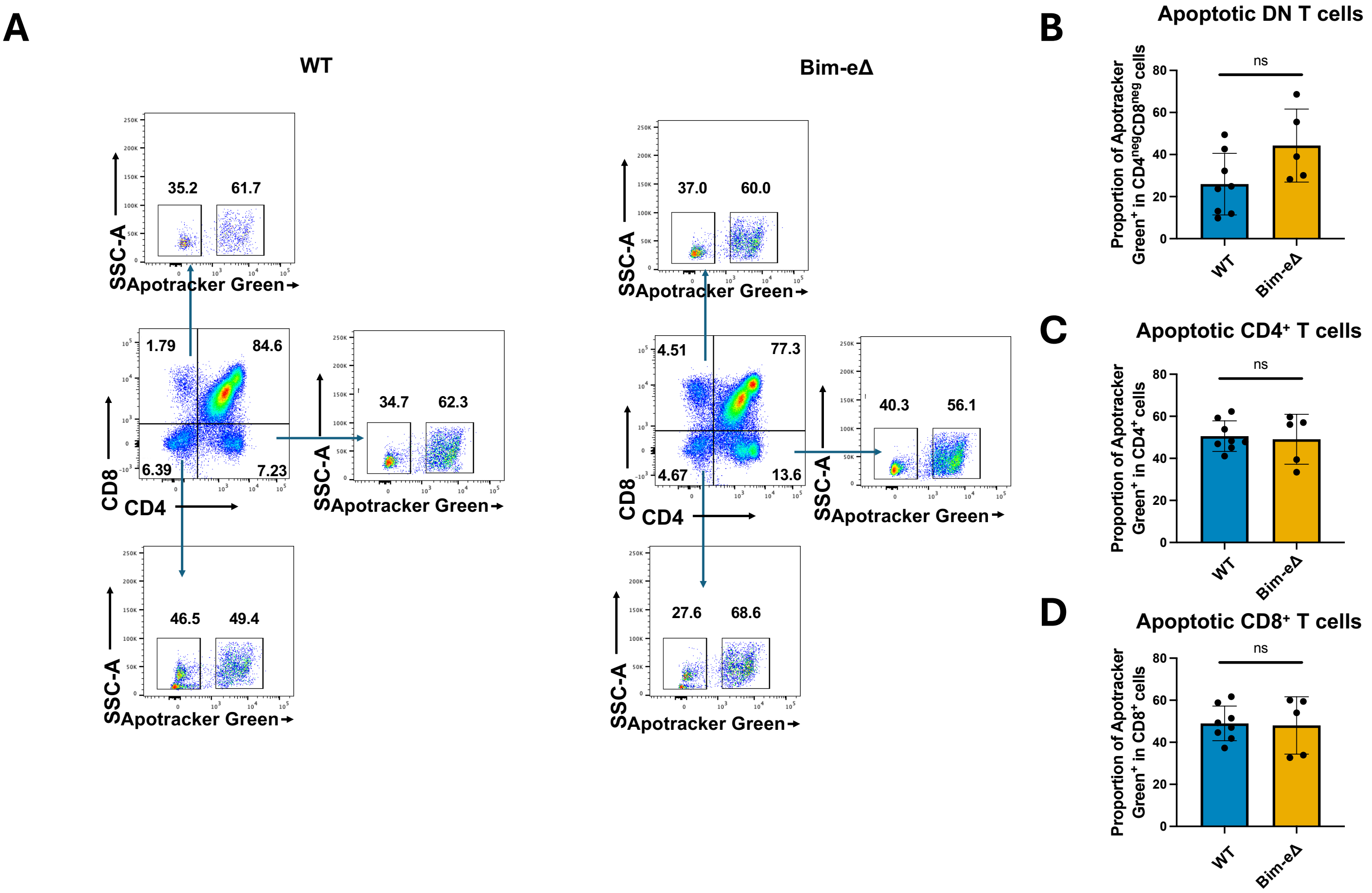

Supplementary Figure 4

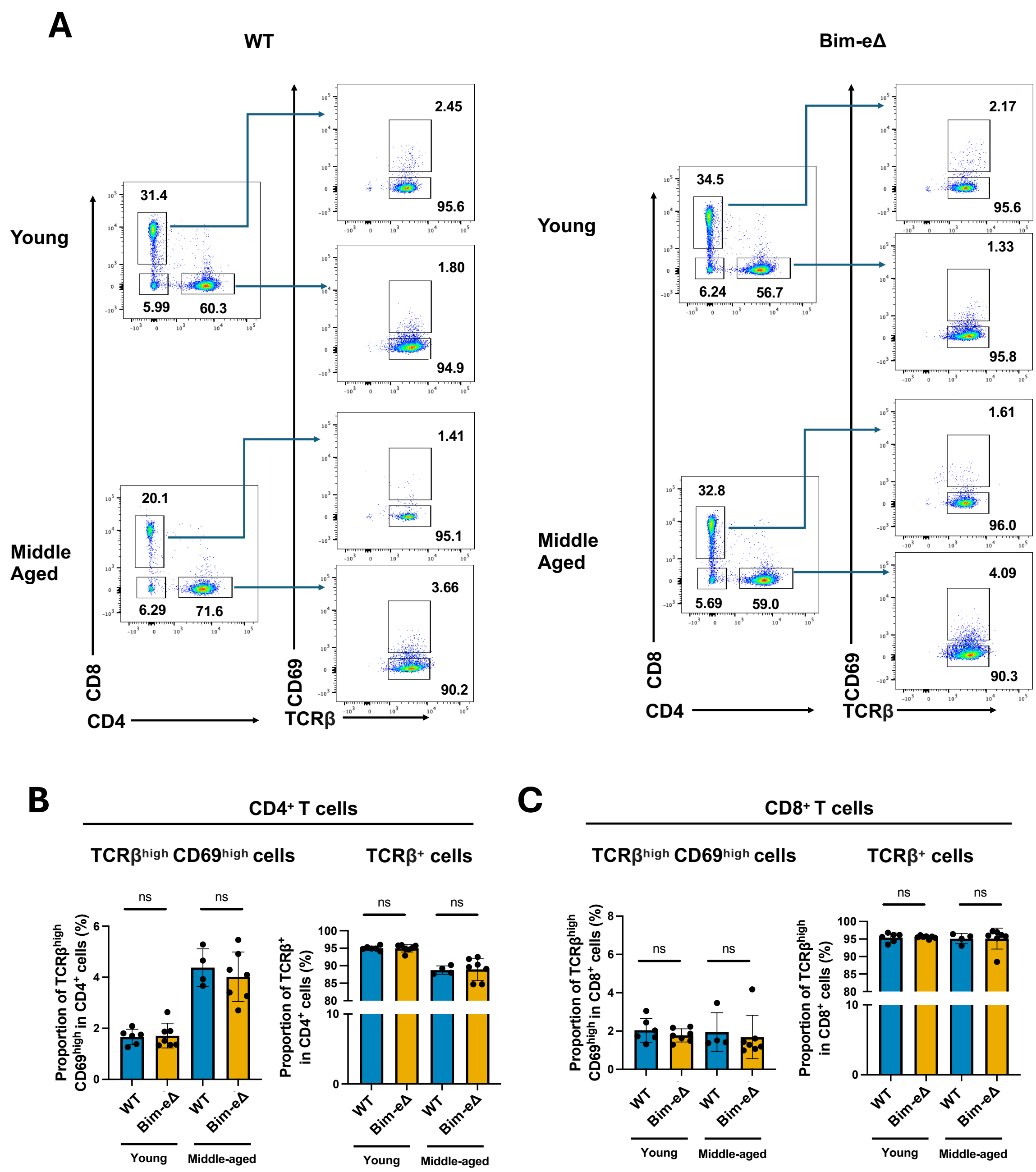

Supplementary Figure 5

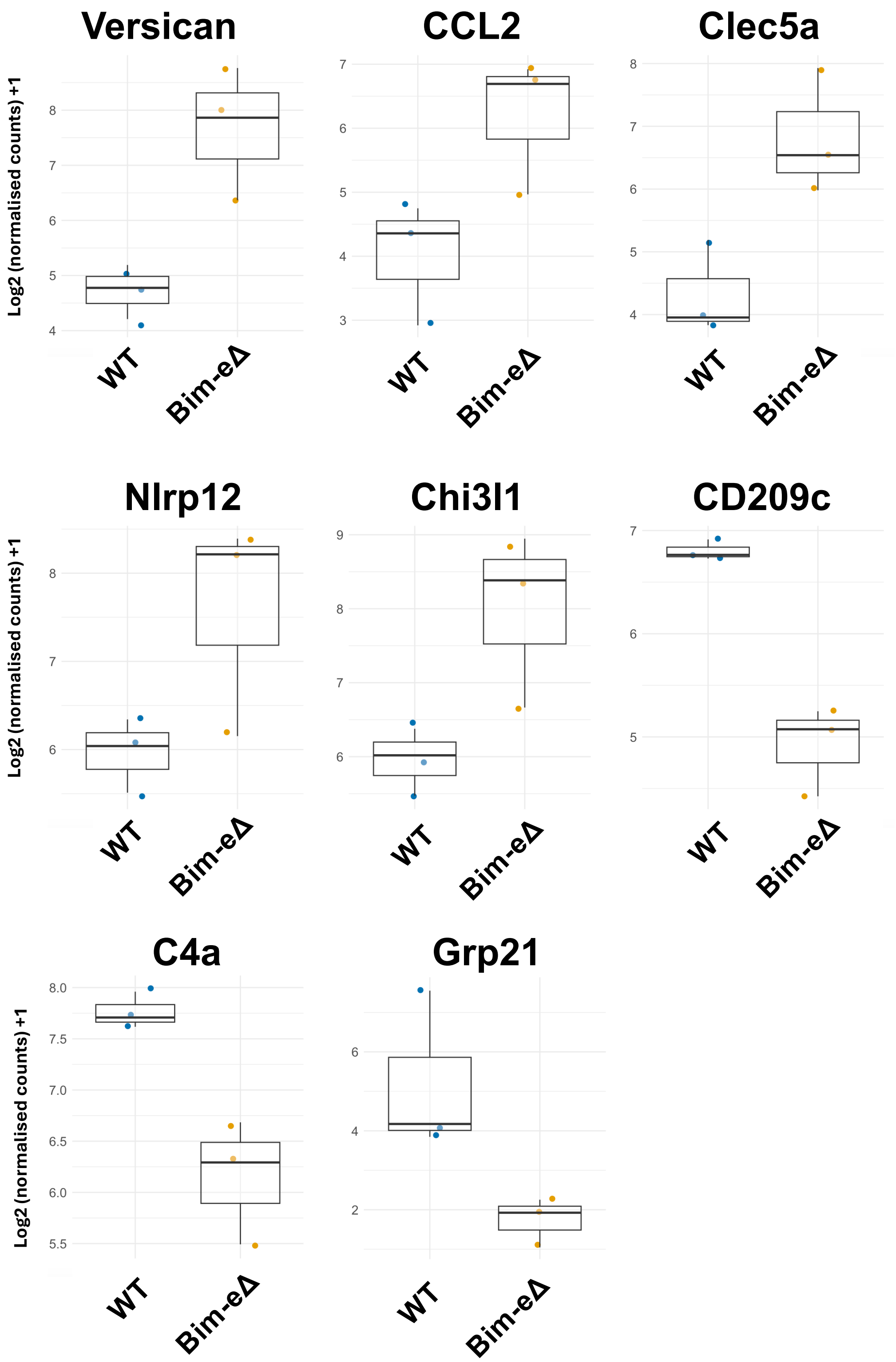

Supplementary Figure 6

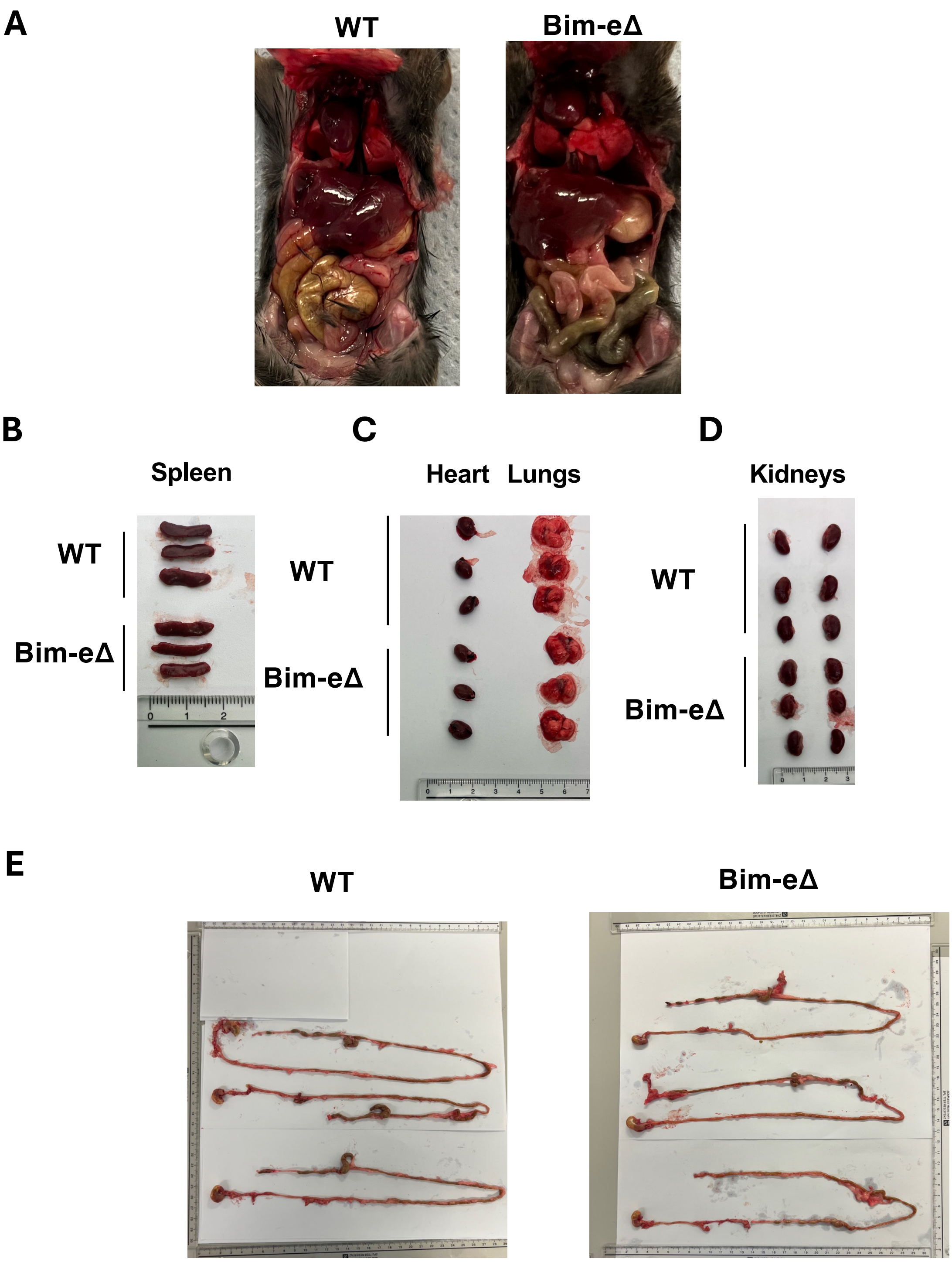

Supplementary Figure 7

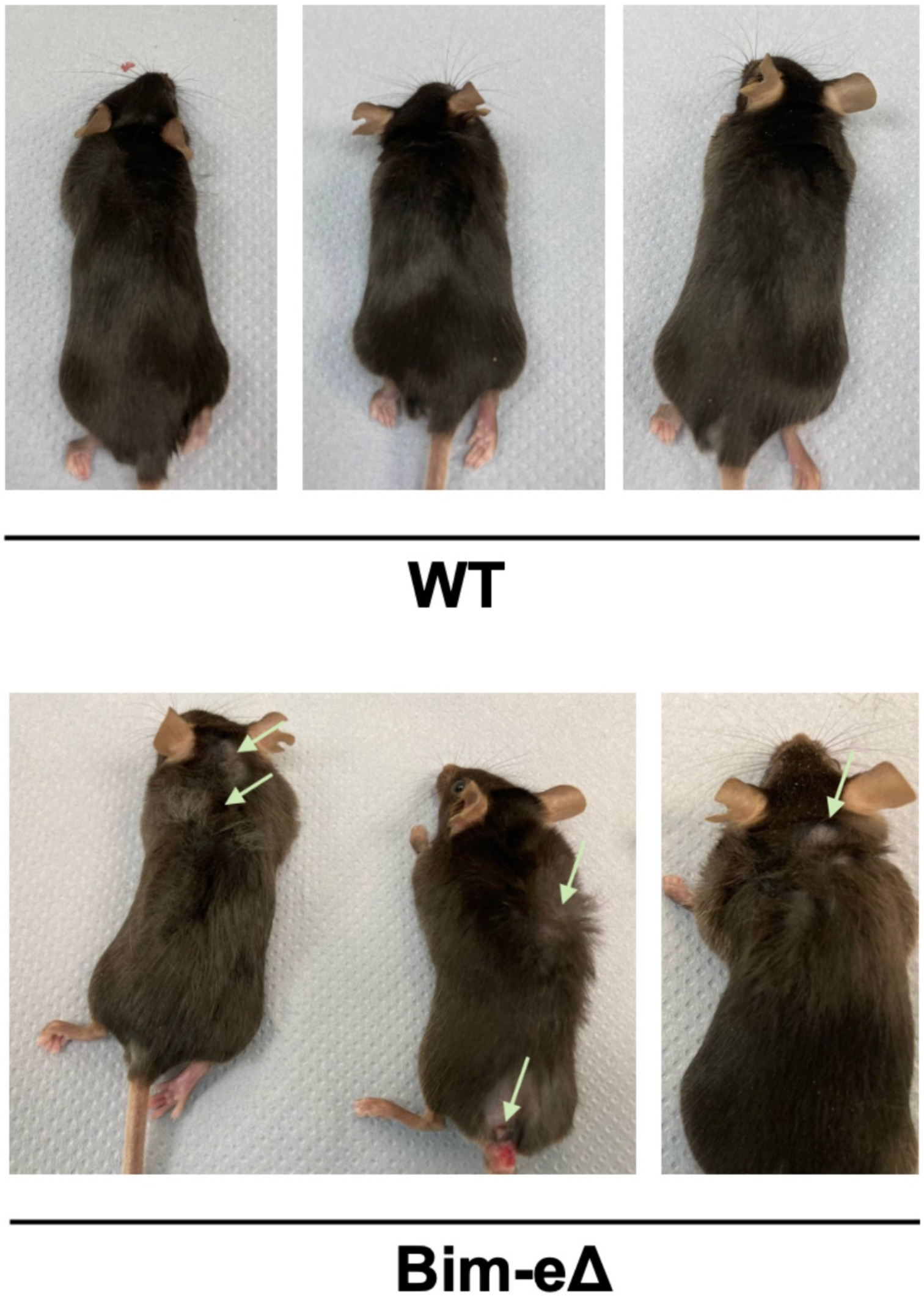

Supplement: Supplementary file 1 [file mmc1.pdf]
